# The enigma of asymptomatic idiopathic pneumoperitoneum: A dangerous trap for general surgeons

**DOI:** 10.1016/j.ijscr.2020.09.149

**Published:** 2020-09-24

**Authors:** M Masood Sidiqi, David Fletcher, Tasfeen Billah

**Affiliations:** Kalgoorlie Regional Hospital, Western Australia, Australia

**Keywords:** Idiopathic, Pneumoperitoneum, Asymptomatic

## Abstract

**Introduction:**

Idiopathic pneumoperitoneum is an extremely rare condition that can easily be misdiagnosed as an acute abdomen. Awareness of this phenomenon can help avoid unnecessary surgical intervention and the potential associated morbidities.

**Presentation of case:**

A 76 year old man presented to hospital with dyspnoea and productive cough and was diagnosed with an infective exacerbation of COPD. He subsequently had a CTPA which showed a significant amount of free intraperitoneal gas in the upper abdomen. He was completely asymptomatic from this with no abdominal pain, distension, or significant rise in inflammatory markers. Of note, he had never had previous abdominal surgery or endoscopic procedures. He showed no signs of peritonism and was closely observed with serial abdominal examinations for 24 h. He subsequently tolerated oral intake and was discharged 8 days after admission.

**Discussion:**

Spontaneous pneumoperitoneum can be due to a variety of intrathoracic, gynaecologic, intra-abdominal and iatrogenic causes. This is a rare case of asymptomatic idiopathic pneumoperitoneum where no clear aetiology or risk factors were found for his free intraperitoneal gas. To the best of our knowledge there have only been two published case reports in the English literature describing idiopathic pneumoperitoneum in a patient that was completely asymptomatic from it.

**Conclusion:**

As a general surgeon it is important to be aware of the non-surgical causes of pneumoperitoneum. Knowledge of this uncommon condition may help reduce the risk of unnecessary laparotomy.

## Highlights

• The aetiology of spontaneous pneumoperitoneum includes a variety of intrathoracic, gynaecologic, intra-abdominal and iatrogenic causes.
• Idiopathic pneumoperitoneum is an extremely rare condition where no clear cause has been found and the patient has no risk factors.
• Knowledge of this rare phenomenon can reduce the negative laparotomy rate in such patients.

## 1 Introduction

The term pneumoperitoneum is defined as the presence of free gas in the peritoneal cavity but outside the viscera. In 90% of cases it indicates a surgical emergency as a result of perforation of an intra-abdominal viscus requiring urgent surgical intervention [1]. The remaining 10% are termed “spontaneous” or “non-surgical” pneumoperitoneum which can be due to a variety of intrathoracic, gynaecologic, intra-abdominal, iatrogenic, and other miscellaneous causes, and can usually be managed conservatively [2]. Idiopathic pneumoperitoneum is an even rarer clinical entity where no clear aetiology has been identified, and both perforated viscus and other known causes of free intraperitoneal gas have been excluded [3]. We report an exceedingly rare case of a man presenting with asymptomatic idiopathic pneumoperitoneum, void of abdominal signs or symptoms and normal inflammatory markers, and no identifiable cause for pneumoperitoneum. To the best of our knowledge there have only been two published case reports in the English literature describing idiopathic pneumoperitoneum in a patient that was completely asymptomatic from it [4,5]. Knowledge of this remarkable phenomenon is very important for all practicing general surgeons as this may help avoid unnecessary surgical intervention and the potential associated morbidities. The work has been reported in line with the SCARE criteria [6].

## 2 Presentation of case

A 76-year-old man presented to ED with a two-week history of progressively worsening dyspnoea and productive cough on a background of mild chronic obstructive pulmonary disease (COPD). He had a history of small cell carcinoma of the lung, treated with chemotherapy and radiotherapy, and was in remission. Of note, he had no prior history of abdominal surgery or endoscopic procedures. His regular medications included salbutamol, tiotropium, aspirin, and thiamine. He did not complain of any abdominal pain or distension. He had a healthy appetite and was opening his bowels normally.

**Fig. 1:**
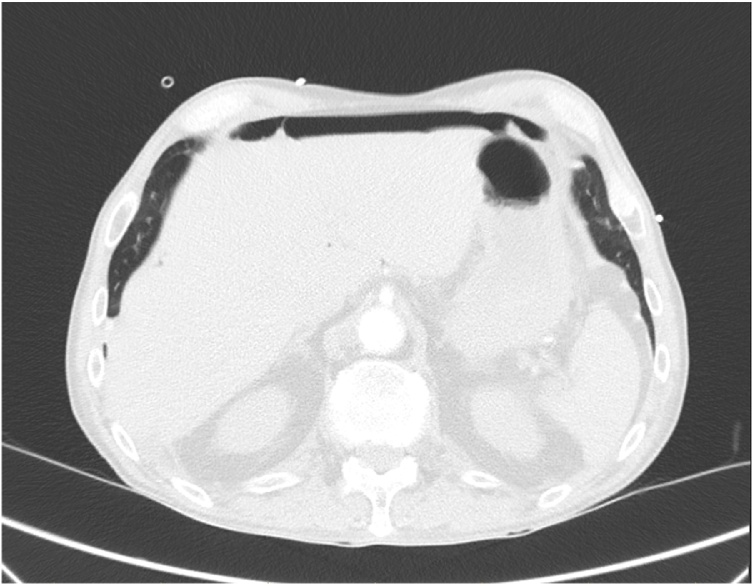
A moderate amount of intraperitoneal gas anterior to the liver.

**Fig. 2:**
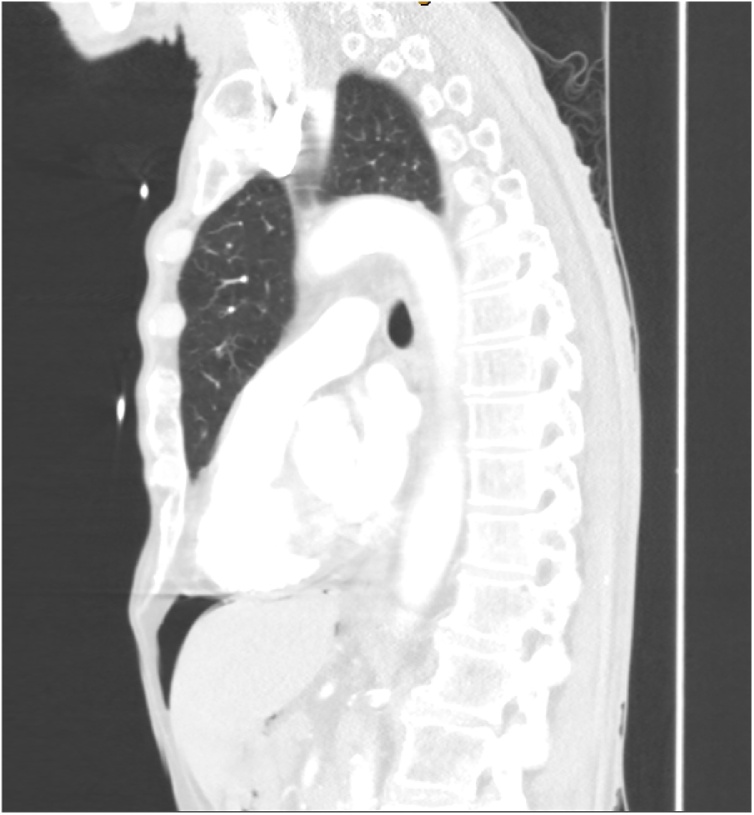
The patient was asymptomatic despite the pneumoperitoneum.

On admission, his oxygen saturations were 94% on 4 L nasal prongs (83% on room air) – all other vital signs were within normal limits and he was afebrile. His abdomen was soft and non-tender, with no apparent distension. There were no signs of peritonism. He had a reducible, non-tender left inguinal hernia. His WCC was 12 × 10^9^/L (>11 × 10^9^/L) and CRP was 10 mg/L (>5.0 mg/L). ECG showed sinus tachycardia and his CXR did not show any obvious consolidation. He was treated as an infective exacerbation of COPD, with antibiotics, bronchodilators, and chest physiotherapy. He subsequently had a CTPA to rule out a pulmonary embolus which showed a moderate amount of intraperitoneal free gas in the upper abdomen (Figs. 1 and 2).

Despite this finding, he was completely asymptomatic with no abdominal pain or distension throughout his hospital admission. His CTPA did not show a pneumothorax or pneumomediastinum, and his recent abdominal CT scans did not show signs of pneumatosis cystoides intestinalis as potential sources of the free air. The general surgical team reviewed the patient and diagnosed him with idiopathic pneumoperitoneum. He was treated conservatively with close observation and serial abdominal examination for the next 24 h. He was allowed oral intake with no clinical deterioration and was discharged on day 8 of his admission. The patient was seen one month later in the outpatient clinics and had remained asymptomatic.

## 3 Discussion

Pneumoperitoneum is classically thought to represent a surgical emergency with gastrointestinal tract perforations accounting for over 90% of cases. There are, however, numerous other causes of pneumoperitoneum that do not require urgent surgical intervention (Table 1). This can present a diagnostic dilemma for the general surgeon confronted with such patients and possibly result in an unnecessary laparotomy.

**Table 1:** The aetiology of spontaneous pneumoperitoneum.

Pneumoperitoneum without peritonitis •Abdominal causes ◦ Post laparotomy/laparoscopy ◦ Pneumatosis cystoides intestinalis/coli ◦ Endoscopy ◦ Peritoneal dialysis/paracentesis •Thoracic causes ◦ Positive pressure ventilation ◦ Pneumomediastinum ◦ Pneumothorax ◦ Barotrauma ◦ Boerhaave syndrome •Gynaecological causes ◦ Pelvic instrumentation (e.g. hysterosalpingography) ◦ Oro-genital intercourse ◦ Vaginal douching ◦ Pelvic inflammatory disease ◦ Pelvic examination (esp. post partum) ◦ Coitus •Rarities ◦ Scuba diving ◦ Jacuzzi usage ◦ Aerophagia

The most common abdominal “non-surgical” cause of pneumoperitoneum is pneumatosis cystoides intestinalis (PCI) [7]. This is a rare and curious condition characterised by submucosal and/or subserosal cysts of free gas in the bowel wall, usually ranging from 0.5 to 2 cm in size. The terminal ileum is the most common location, but it can be found in other sites including the mesentery, omentum, and even the large bowel (pneumatosis cystoides coli) [8]. These cysts can cause pneumoperitoneum and peritoneal irritation either by rupture or diffusion [9]. It is important to note that rupture of these cysts do not represent a true bowel perforation through all layers of the bowel wall, as they are only submucosal and subserosal, and do not communicate with the lumen of the bowel. PCI is most commonly idiopathic, but can also be associated with connective tissue diseases. There have been reports of it being associated with chemotherapy, hormonal therapy, inflammatory bowel disease, and even organ transplant [10,11]. Treatment is typically supportive with supplemental oxygen, and most cases follow a benign course and resolve spontaneously [12]. It is important to differentiate PCI with a secondary form of this condition known as pneumatosis intestinalis which is not in fact a disease, but a radiological finding occurring as a consequence of acute gastrointestinal ischaemia.

Tracking of air from the mediastinum into the retroperitoneum and peritoneal cavity is also a well-recognised phenomenon. Intrathoracic causes of pneumoperitoneum include thoracic trauma (such as barotrauma), pneumothorax, cardiopulmonary resuscitation, and invasive ventilation, particularly in cases with high peak inspiratory pressures. In such cases raised intrathoracic pressure leads to leakage of intrathoracic air through microscopic pleural and diaphragmatic defects, or retroperitoneal perivascular sheaths, and pneumomediastinum often co-exists [5,13].

The female genital tract can also provide a portal of entry for air into the peritoneal cavity in certain conditions. During laparoscopic investigation for infertility, dye insufflated via the external os can be seen emerging from the fimbrial ends of a patent salpinx [2]. As a result of this anatomical communication between the peritoneal cavity and the fallopian tubes and endometrium, air may enter the peritoneum [14]. Sexual intercourse, especially after hysterectomy, has been reported to cause spontaneous pneumoperitoneum [15]. Oral-genital insufflation, vaginal douching, pelvic inflammatory disease, and even knee-chest exercises post-partum have also been reported [2,14,16].

The most common iatrogenic cause of pneumoperitoneum is following abdominal surgical procedures (laparotomy and laparoscopy), and in the majority of cases the air is completely resorbed within two weeks after the procedure [2]. There have also been cases of benign pneumoperitoneum post routine diagnostic colonoscopy and therapeutic colonoscopy with argon plasma coagulation [[17], [18], [19]]. Transmural passage of insufflated gas without bowel wall compromise was thought to be the mechanism in these cases. Subclinical visceral microperforation has also been proposed in such cases, with a valve-like arrangement permitting the escape of air but inhibiting the leakage of liquid contents with changes in the pressure of the gastrointestinal tract [20,21]. Other bizarre reports of spontaneous pneumoperitoneum have been documented after jacuzzi usage, aerophagia, scuba diving, and even dental extraction [2,[22], [23], [24]].

Finally, there exists a group of patients with pneumoperitoneum who have no clear aetiology or demonstrable risk factors; these are true cases of idiopathic pneumoperitoneum [2]. The majority of these cases present with a variety of symptoms including abdominal pain, distension, fever, and raised inflammatory markers, but without clinical signs of peritonitis [1,9,19,20,25,26]. Our patient is an exceedingly rare case of idiopathic pneumoperitoneum who was completely asymptomatic from the abdominal point of view. Such patients pose a significant challenge to the clinician in regards to investigation and management, and given the initial concern of perforated viscus, many of them may undergo negative laparotomy exposing them unnecessarily to the risk of various surgical and anaesthetic complications. Thus, it is important to have a systematic approach for identifying which patients need urgent surgical intervention and which can be managed conservatively. A detailed history and examination is critical in distinguishing surgical and non-surgical pneumoperitoneum. The presence or absence of peritonitis on clinical examination and determining the underlying cause of pneumoperitoneum also play a vital role in deciding management. Identifying an underlying cause is best achieved by contrast-enhanced CT which can predict the location of a potential perforation with 86% accuracy [27]. If the patient does not exhibit signs of peritonitis, conservative management with close observation may suffice [[28], [29], [30]]. Treatment methods such as intravenous antibiotics, total parenteral nutrition, bowel rest, serial examinations, and repeat imaging have all been documented in the literature with success [5,25,26]. It must be noted that clinical deterioration always presents a potential indication for surgical intervention. In summary, if clinical features of peritonitis are absent, a careful history and search for radiological evidence of pneumatosis cystoides, pneumothorax, pneumomediastinum or retroperitoneal air may indicate a benign cause and reduce the risk of unnecessary laparotomy.

## 4 Conclusion

Idiopathic pneumoperitoneum is an extremely rare condition and can easily be misdiagnosed as an acute abdomen. It is a diagnosis of exclusion which should only be made once surgical and nonsurgical causes have been ruled out. We report a rare case of asymptomatic idiopathic pneumoperitoneum that was successfully managed conservatively with only serial abdominal examinations. It is hoped that greater awareness of this condition and a discerning approach to its management will help reduce the negative laparotomy rate in such patients.

## Declaration of Competing Interest

The authors report no declarations of interest.

## Funding

None.

## Ethical approval

Ethical approval is not applicable.

## Consent

Written informed consent was obtained from the patient for publication of this case report and accompanying images. A copy of the written consent is available for review by the Editor-in-Chief of this journal on request.

## Author contribution

Dr Masood Sidiqi and Tasfeen Billah (medical student) contributed in medical record review, literature search, and writing of the draft. Dr David Fletcher contributed towards review of the paper.

## Registration of research studies

N/A.

## Guarantor

Dr Masood Sidiqi.

## Provenance and peer review

Not commissioned, externally peer-reviewed.
